# Analysis methods for studying the 3D architecture of the genome

**DOI:** 10.1186/s13059-015-0745-7

**Published:** 2015-09-02

**Authors:** Ferhat Ay, William S. Noble

**Affiliations:** Department of Genome Sciences, University of Washington, Seattle, WA, 98195 USA; Feinberg School of Medicine, Northwestern University, Chicago, 60661 IL USA; Department of Computer Science and Engineering, University of Washington, Seattle, 98195 WA USA

**Keywords:** Genome architecture, Chromatin conformation capture, Three-dimensional genome, Three-dimensional modeling

## Abstract

The rapidly increasing quantity of genome-wide chromosome conformation capture data presents great opportunities and challenges in the computational modeling and interpretation of the three-dimensional genome. In particular, with recent trends towards higher-resolution high-throughput chromosome conformation capture (Hi-C) data, the diversity and complexity of biological hypotheses that can be tested necessitates rigorous computational and statistical methods as well as scalable pipelines to interpret these datasets. Here we review computational tools to interpret Hi-C data, including pipelines for mapping, filtering, and normalization, and methods for confidence estimation, domain calling, visualization, and three-dimensional modeling.

## Introduction

Now, more than ever, it is recognized that the three-dimensional organization of chromatin affects gene regulation and genome function. Capturing chromosome conformation, first at the level of single locus (3C, 4C) [1–4] or a set of loci (5C, ChIA-PET) [5, 6], and then genome-wide (Hi-C) [7–9], made it possible to link chromatin structure to gene regulation [10–18], DNA replication timing [19–21], and somatic copy number alterations [22, 23]. Furthermore, genome-wide conformation capture studies reveal conserved structural features that are now accepted as organizing principles of chromatin folding [7, 15, 18, 24]. Hi-C data have also proved to be useful in many other applications, ranging from genome assembly and haplotyping [25–27] to finding the coordinates of centromeres and ribosomal DNA (rDNA) [28, 29]. See [7–9, 18, 24, 30] for detailed descriptions of how the Hi-C assay and its variants work. Briefly, the traditional Hi-C assay consists of six steps: (1) crosslinking cells with formaldehyde, (2) digesting the DNA with a restriction enzyme that leaves sticky ends, (3) filling in the sticky ends and marking them with biotin, (4) ligating the crosslinked fragments, (5) shearing the resulting DNA and pulling down the fragments with biotin, and (6) sequencing the pulled down fragments using paired-end reads. This procedure produces a genome-wide sequencing library that provides a proxy for measuring the three-dimensional distances among all possible locus pairs in the genome.

We discuss below the processing pipelines, tools, and methodologies for analysis of Hi-C data. Understanding how these Hi-C analysis methods work and the available options to perform each analysis step is becoming more important with the increasing number and variety of Hi-C datasets. Currently, Hi-C data are available for a wide variety of organisms, such as yeasts [8, 28, 31–33], bacteria [34], fruit fly [30, 35, 36], plants [37–39], malarial parasites [16, 40], and numerous human and mouse cell lines [7, 15, 18, 24, 41–44].

### Mapping, filtering, and classification of Hi-C reads

The initial processing step for Hi-C data typically consists of trimming of reads (if necessary), mapping the reads to the corresponding reference genome with assay-specific pre- and post-processing to improve the percent of mapped reads, and filtering of the mapped reads and read pairs at several different levels. We outline below the details of several mapping and filtering approaches used for Hi-C data. Note that, to distinguish between single-end and paired-end reads, we will refer to them as ‘reads’ and ‘read pairs’, respectively.

### Mapping

The two ends of a paired-end Hi-C read ideally correspond to locations that are far apart along the genome. In other words, most sequence fragments in a high-quality Hi-C library are composed of DNA from two or more non-contiguous loci. Such fragments are referred to as chimeras. When the two ends of a long chimeric fragment are sequenced, if the ligation junction falls near the middle of the fragment, then each of the resulting reads will map to a different location in the genome. However, if the ligation junction happens to fall within one of the sequenced ends of the fragment, then the read itself will be chimeric. Furthermore, if the parent fragment is a chimera involving more than two genomic loci, then both reads can potentially be chimeric. The frequency of such chimeric reads depends heavily on several factors, including the size-selection step and the read length used for sequencing [18, 45].

Partly as a result of this dependence and partly because of interpretation differences, there are now many proposed ways to handle mapping of Hi-C reads. The simplest approach is to filter out any read that does not fully map to the genome because it is chimeric. This approach may be acceptable when size-selected fragments are very long (800 bp) and read length is relatively short (50 bp) [30]. However, shorter fragment lengths and longer reads are more commonly used in Hi-C experiments. For instance, using the 4-cutter restriction enzyme MboI, size selecting for 300–500 bp fragments and sequencing with 101 bp reads leads to approximately 20 % of sequenced read pairs with at least one chimeric end [18]. We are aware of at least four different ways to ‘rescue’ information from such chimeric Hi-C reads. Two of these alternatives pre-process reads before initial mapping and the other two post-process the results after an initial attempt to map all reads at their full lengths. Instructions for these methods are as follows.

**Fig. 1:**
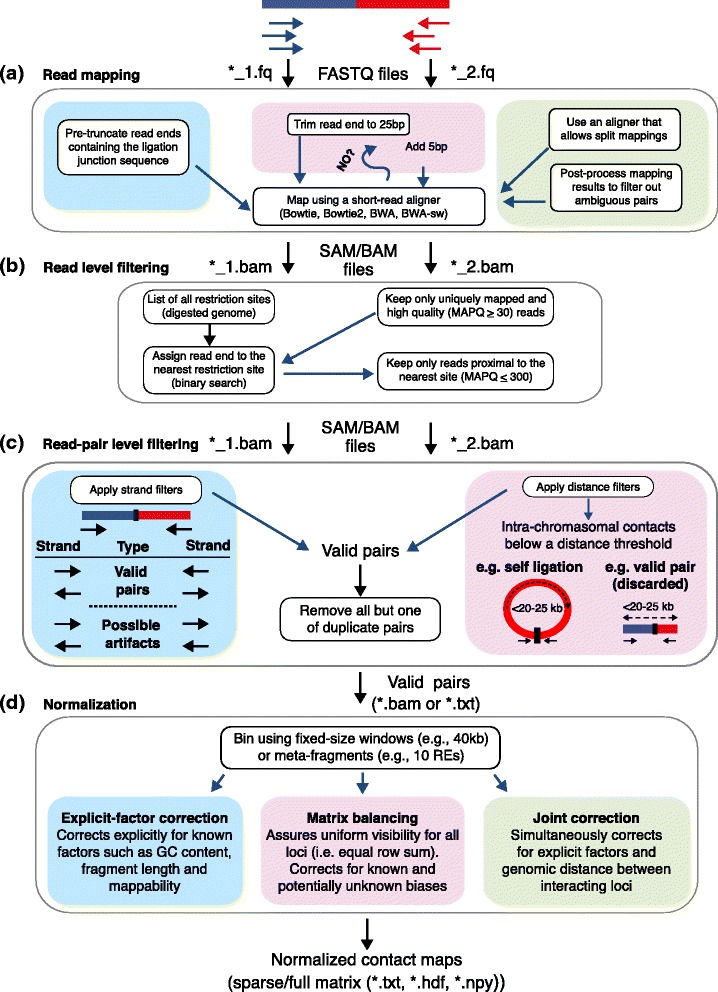
Overview of Hi-C analysis pipelines. These pipelines start from raw reads and produce raw and normalized contact maps for further interpretation. The colored boxes represent alternative ways to accomplish a given step in the pipeline. RE, restriction enzyme. At each step, commonly used file formats (‘.fq’, ‘.bam’, and ‘.txt’) are indicated. **a**, The blue, pink and green boxes correspond to pre-truncation, iterative mapping and allowing split alignments, respectively. **b**, Several filters are applied to individual reads. **c**, The blue and pink boxes correspond to strand filters and distance filters, respectively. **d**, Three alternative methods for normalization

**Pre-truncation:** Pre-process all the reads and truncate the ones that contain potential ligation junctions to keep the longest piece without a junction sequence [46] (Fig. 1a, blue box). For restriction enzymes that leave sticky ends, the ligation junction sequence is a concatenation of two filled-in restriction sites (for example, AAGCTAGCTT for HindIII that cuts at A |AGCTT and GATCGATC for MboI that cuts at GATC |).

**Iterative mapping:** Trim the reads to only keep the 25 bp-long 5 ^′^ portion. If this portion fails to map uniquely then repeat the mapping attempt by adding 5 bp to the read at each iteration until the full read length is reached (Fig. 1a, pink box) [47].

**Allow split alignments:** For mapping use a short-read aligner that allows split alignments within a read (such as BWA’s *bwa-sw* mode [48]). Identify reads that fully align and that align in split mode and post-process the latter category to only keep the ‘unambiguous’ read pairs that have one end mapping to two loci A and B and the other end mapping to either A or B (Fig. 1a, green box) [18].

**Split if not mapped:** Attempt to map all the reads at their full lengths using the regular mode of an aligner (such as BWA’s *aln* [48] or Bowtie [49]). Among the non-mapped reads, identify the ones containing exactly one restriction site, break such reads into two pieces and map each piece independently back to the genome. This approach allows the identification of simultaneous contacts among three or four loci, which can then be broken into pairs [45]. Note that the search for a restriction site is valid only for protocols that skip the end repair step or use a blunt end restriction enzyme (such as AluI that cuts at AG |CT). For traditional Hi-C libraries, this step needs to be replaced by a search for the ligation junction sequence.

### Read-level filtering

Once the individual reads are mapped to the genome, the next step is to decide which of these mapped reads to ‘trust’. The first step is to apply standard filters on the number of mismatches (usually none allowed), mapping quality (MAPQ score), and uniqueness of the mapped reads, similar to any other sequencing-based assay. The second step is to create a list of all possible restriction sites (not to be confused with ligation junction sequences) in the reference genome and to assign each read to the nearest restriction site. It is important to note that the number of restriction sites can be high (for the human genome > 800,000 and > 7 million for HindIII and MboI, respectively), necessitating the use of scalable methods such as binary search to find the nearest restriction site for each read. In the third step, the distance between each read’s start coordinate and the nearest restriction site is used to filter out reads that do not agree with the size-selection step (Fig. 1b).

### Read-pair level filtering

In most Hi-C pipelines, read pairs for which both ends successfully pass through the initial filters are further segregated into several categories. The aim of this classification is to identify and proceed further with only the pairs that provide information about three-dimensional chromatin conformation beyond linear proximity among regions. We will refer to these as ‘informative pairs’. These read-pair level filtering approaches can be categorized into two main groups, strand and distance filters (Fig. 1c). Many Hi-C pipelines use a combination of the two approaches to ensure stringent filtering of all possible artifacts.

**Strand filters:***De novo* ligations introduced by the Hi-C protocol should have no preference for a specific strand combination or orientation and result in paired-end reads with each end coming from a different restriction fragment. Figure 2 of Lajoie et al. [50] provides a detailed description of all possible orientation combinations arising from Hi-C read mapping. Briefly, there are two main cases: either the read pair falls within the same restriction fragment or in two distinct restriction fragments. Regardless of the strand combination, a read pair coming from a single restriction fragment is uninformative of chromatin conformation and should be filtered out. For the second case, in which a read pair links two distinct fragments, Fig. 1c illustrates all possible strand combinations. In this case, if two read ends either point towards (inward orientation (+/−)) or away from each other (outward orientation (−/+)), the corresponding pair is a valid pair that is informative of chromatin conformation. The remaining same-strand pairs (+/+ or −/−) could either be valid pairs or artifacts that come from undigested chromatin. Such pairs from undigested chromatin will correspond to a distance between the two mapping coordinates that is small and consistent with the size of fragments that are selected by the size-selection step. Detailed analyses of strand-related biases suggest filtering inward and outward pairs separated by < 1 kbp and < 25 kbp distances, respectively [42]. Several recently published studies [18, 42] provide metrics to quantify strand-related biases and suggest additional filtering schemes for processing high-resolution Hi-C data.

**Fig. 2:**
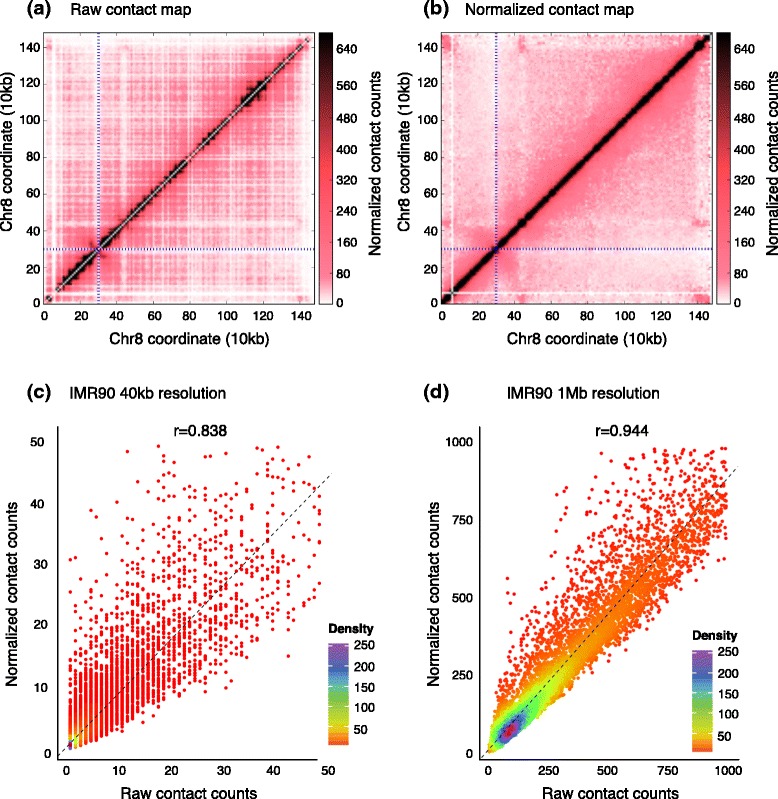
Impact of normalization on Hi-C contact maps. **a**, **b** Hi-C contact maps of chromosome 8 from the schizont stage of the parasite *Plasmodium falciparum* [16] at 10 kb resolution before and after normalization. Blue dashed lines represent the centromere location. **c**, **d** Density scatter plots of counts before (x-axis) and after (y-axis) normalization of Hi-C data from the human cell line IMR90 [15] at two different resolutions. Correlation values are computed using all intra-chromosomal contacts within human chromosome 8. Only a subset of points are shown for visualization purposes

**Distance filters:** Most, if not all, of the read pairs discarded by strand filters are intra-chromosomal pairs separated by short genomic distances. Therefore, one way to achieve read-pair level filtering is to simply filter out intra-chromosomal pairs below a certain distance threshold. This distance-based filter was widely used for earlier Hi-C data because it is fairly effective for low resolution Hi-C studies [7, 8]. The distance threshold is empirically set at 20–25 kb or larger. However, this approach discards, in addition to artifacts such as self ligation products or undigested chromatin, potentially interesting contacts occurring within this distance range (Fig. 1c). Another downside of a simple distance threshold is its inability to detect certain artifacts, such as self-ligations for very long (> 25 kb) fragments.

One last filtering step is the identification and removal of duplicated read pairs. Because reads produced by standard Hi-C assays come from a population of cells, these duplicates may indeed be valid read pairs from different cells or PCR duplicates of a read pair from one single cell. Lacking a method to distinguish between these two cases, current practice is to simply discard all but one pair from a set of duplicates. This approach avoids any potential PCR artifacts at the expense of losing some potentially informative read counts. However, because of the high complexity of Hi-C libraries, the duplicate percentage is generally very low. Duplicate removal can be carried out by Picard [51] or a simple shell script.

Table 1 summarizes currently available Hi-C tools and pipelines, and indicates which processing steps can be performed with each tool. Comprehensive and up-to-date lists of these tools are available from Omictools [52] and the Structural Genomics group at CNAG, part of the Spanish Center for Genomic Regulation [53]. Some of these tools focus more on the initial steps such as mapping and filtering (such as HiCUP and HiC-inspector), whereas others focus on downstream analysis tasks such as normalization, visualization, and statistical confidence estimation. The latter tasks are described below.

**Table 1:** Software tools for Hi-C data analysis

Tool	Short-read	Mapping	Read	Read-pair	Normalization	Visualization	Confidence	Implementation
	aligner(s)	improvement	filtering	filtering			estimation	language(s)
HiCUP [46]	Bowtie/Bowtie2	Pre-truncation	✓	✓	−	−	−	Perl, R
Hiclib [47]	Bowtie2	Iterative	✓ ^*a*^	✓	Matrix balancing	✓	−	Python
HiC-inspector [131]	Bowtie	−	✓	✓	−	✓	−	Perl, R
HIPPIE [132]	STAR	✓ ^*b*^	✓	✓	−	−	−	Python, Perl, R
HiC-Box [133]	Bowtie2	−	✓	✓	Matrix balancing	✓	−	Python
HiCdat [122]	Subread	−^*c*^	✓	✓	Three options ^*d*^	✓	−	C++, R
HiC-Pro [134]	Bowtie2	Trimming	✓	✓	Matrix balancing	−	−	Python, R
TADbit [120]	GEM	Iterative	✓	✓	Matrix balancing	✓	−	Python
HOMER [62]	−	−	✓	✓	Two options ^*e*^	✓	✓	Perl, R, Java
Hicpipe [54]	−	−	−	−	Explicit-factor	−	−	Perl, R, C++
HiBrowse [69]	−	−	−	−	−	✓	✓	Web-based
Hi-Corrector [57]	−	−	−	−	Matrix balancing	−	−	ANSI C
GOTHiC [135]	−	−	✓	✓	−	−	✓	R
HiTC [121]	−	−	−	−	Two options ^*f*^	✓	✓	R
chromoR [59]	−	−	−	−	Variance stabilization	−	−	R
HiFive [136]	−	−	✓	✓	Three options ^*g*^	✓	−	Python
Fit-Hi-C [20]	−	−	−	−	−	✓	✓	Python

^a^Hiclib keeps the reads with only one mapped end (single-sided reads) for use in coverage computations

^b^HIPPIE states that it rescues chimeric reads. No details are given

^c^HiCdat reports no substantial improvement in successfully aligned read pairs when iterative mapping in Hiclib is used for *Arabidopsis thaliana* Hi-C data

^d^HiCdat provides three options for normalization: coverage and distance correction, HiCNorm and ICE

^e^HOMER provides two options for normalization: simpleNorm corrects for sequencing coverage only and norm corrects for coverage plus the genomic distance between loci

^f^HiTC provides two options for normalization: normLGF implements HiCNorm and normICE implements ICE algorithm from Hiclib

^g^HiFive provides three options - Probability, Express, and Binning - for normalization. The Express and Binning algorithms correspond to matrix balancing and explicit-factor correction schemes, respectively

## Normalization of Hi-C contact maps

Not long after the first Hi-C datasets became available [7, 8], several sequence-dependent features were shown to substantially bias Hi-C readouts [54]. These include biases that are associated with sequencing platforms (such as GC content) and read alignment (such as mappability), and those that are specific to Hi-C (such as frequency of restriction sites). Discovery of these biases led to several normalization or correction methods for Hi-C data [47, 54–59].

Before discussing these methods, it is necessary to describe how the data are represented in matrix form. A contact map is a matrix with rows and columns representing non-overlapping ‘bins’ across the genome. Each entry in the matrix contains a count of read pairs that connect the corresponding bin pair in a Hi-C experiment. These bins can be either fixed-size genomic windows or can correspond to a fixed number of consecutive restriction fragments (Fig. 1d). The binning step consists of determining the binning type (fixed-size or restriction-fragment-based) and bin size that is appropriate given the sequencing depth in hand, assigning each valid pair that passed all filters to a specific bin pair, and incrementing the count in the corresponding matrix entry. Determining the appropriate bin size is an important task and involves a tradeoff between resolution and statistical power. Several published studies use multiple bin sizes to analyze a single set of Hi-C data. Even though there are no clear guidelines yet, a recent study suggests using a bin size that results in at least 80 % of all possible bins having more than 1,000 contacts [18]. According to this criterion, approximately 300 million mapped reads are needed to achieve 10 kb resolution for the human genome, assuming that all reads are uniformly distributed across the genome. However, this criterion suggests a linear relationship between resolution and sequencing depth, which does not hold for two-dimensional Hi-C data. An alternative would be to use a similar cutoff-based measure on the density of either the *cis*- or the *trans*-contact matrices instead of total contact counts per locus. Once the bin size is determined and the binning is done, the resulting raw contact map Fig. 2a serves as the input for the normalization methods described below.

### Explicit-factor correction

Normalization methods of this type require *a priori* knowledge of the factors that may cause bias in Hi-C data. Yaffe and Tanay identify three such factors and develop a joint correction procedure that models the probability of observing a contact between two regions given their genomic features, such as GC content, mappability, and fragment length that are shown to affect contact counts [54]. A later method, HiCNorm [55], provides a significantly faster explicit correction method by using regression-based models (either negative binomial or Poisson regression) while achieving similar normalization accuracy to that of the Yaffe and Tanay method.

### Matrix balancing

Another approach to normalization is to correct for all factors that may cause biases without explicitly modeling them. Methods of this type rely on the important assumption that if there were no bias then each locus in the genome would be ‘equally visible’ or, in other words, give rise to an equal number of reads in a Hi-C experiment. This assumption, of which we will later discuss the ramifications, transforms the normalization to a matrix balancing problem where the aim is to find a decomposition of the observed contact map $O=\vec {b}^{T}$*T*$\vec {b}$ such that $\vec {b}$ is a column vector of bias terms and *T* is a normalized contact map in which all rows have equal sums. This matrix balancing problem has been studied for several decades in many different contexts (see the Supplemental Information of [18] for a detailed discussion). In the context of Hi-C, Imakaev et al. proposed an iterative method abbreviated as ICE [47], which applies a previously described algorithm [60] repeatedly to achieve the desired decomposition. Cournac et al. also proposed a very similar iterative correction method for Hi-C data, which they named Sequential Component Normalization. More recently, Rao et al. [18] used a much faster matrix balancing algorithm by Knight and Ruiz [61] to normalize their high-resolution Hi-C datasets sequenced using billions of reads. Development of scalable and memory-efficient tools for normalizing high-resolution Hi-C contact maps using matrix balancing is still an ongoing effort [57].

### Joint correction

The strongest determinant of how many contacts are observed between a pair of regions on the same chromosome is the genomic (one-dimensional) distance between them. This is an unsurprising outcome of polymer looping, which dictates that regions adjacent to each other in one dimension cannot be far away in three-dimensional space. Although many methods consider this polymer looping effect later in the Hi-C data analysis [18, 20, 62], some others jointly ‘normalize’ for this one-dimensional distance effect during the normalization for the above mentioned biases. For instance, GDNorm extends the Poisson regression framework of HiCNorm to include spatial (three-dimensional) distances in normalization, which the method achieves by restricting the space of possible three-dimensional distances using genomic or one-dimensional distance information [58]. In other work, Jin et al. [42] adapt Yaffe and Tanay’s method [54] to correct for both the biases pointed out by the original method and also for the genomic distance between two loci on the same chromosome that are at most 2 Mb apart.

Overall, these studies show that normalization is essential for Hi-C data. Normalized contact maps are visually smoother than their raw versions, making it easier to spot potentially interesting contact patterns (Fig. 2a, b). Furthermore, normalization significantly improves the reproducibility between replicates of a Hi-C library created with two different restriction enzymes [47, 54, 55, 59]. In general, the raw and normalized contact counts are highly correlated for low resolution data. However, this correlation drops with increasing resolution, suggesting that normalization is even more important for high-resolution Hi-C datasets (Fig. 2c, d).

Even though several different normalization methods produce highly similar outputs [47, 55], each normalization method requires invoking some debatable assumptions. For instance, explicit-factor correction methods assume that only a predetermined set of biases exist in the data and that these biases can be corrected using a single-step visibility correction [54, 55]. In contrast, matrix balancing methods aim to eliminate all biases, known or unknown, through an iterative correction of visibility that leads to a uniform coverage of each fixed-size genomic window. However, the assumption that ‘equal visibility equals no bias’ can be problematic when certain regions have mappability issues or are inherently limited in their ability to form long-range contacts [63, 64]. To alleviate these issues, a pre-filtering step for loci with very low visibility and a post-normalization visual inspection is usually necessary to avoid occasional artifacts from matrix balancing-based methods [47, 50].

Aside from these limitations, most current implementations of the normalization methods discussed here cannot directly handle high-resolution human Hi-C data below 10 or 50 kb resolution without using parallel computing or graphics processing units (GPUs), which are more powerful than standard central processing units (CPUs) [18, 57].

## Extracting significant contacts

A unique aspect of chromatin conformation capture data is that it enables us to search for long-range contacts, either between locus pairs that are on the same chromosome but far from each other (long-range intra-chromosomal) or on different chromosomes (inter-chromosomal). Identifying statistically significant inter-chromosomal contacts is straightforward because, once biases are eliminated by normalization, in the absence of any prior information on the pairwise distances among chromosomes, all possible pairs of inter-chromosomal loci are expected to interact equally under the null hypothesis. However, the number of contacts between two intra-chromosomal loci depends heavily on the genomic distance between the loci. This dependence is mainly due to random looping of the DNA rather than formation of specific chromatin loops. Therefore, one needs to control for this random polymer looping when assigning statistical significance to the observed contact counts. Below we outline several approaches to significance estimation that take into account the distance dependence of contact counts.

### Observed/expected ratio

One way to account for the distance dependence of contact counts is to bin together all pairs of loci with the same or similar genomic distances. Earlier Hi-C and 5C processing methods used this approach to compute a ratio [7], a *p*-value [8] or a *z*-score [65] for each contact count with respect to the average number of contacts within a genomic distance bin. Using a similar approach, more recent methods create background models of contact counts that take into account the distance scaling, domain organization and other biases corrected by the normalization methods [30, 62]. These background models are then used to compute observed/expected ratios that are either subjected to ad hoc enrichment cutoffs or are transformed to *p*-values or *z*-scores.

### Parametric fits

Another approach is to assume that a specific distribution captures the distance dependence of contact counts and to perform parameter estimation to find the best fit to the data. Previously used distributions include power-law [7], double-exponential [31], and negative binomial [42]. Once a parametric fit to the data is found, these methods compute either an enrichment score or statistical significance for each locus pair using their genomic distance and their contact count.

### Nonparametric fits

**Fig. 3:**
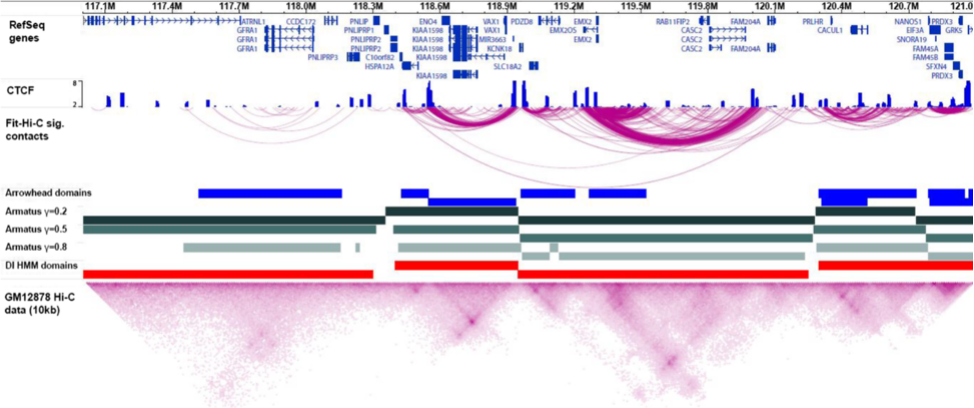
Visualization of Hi-C data. An Epigenome Browser snapshot of a 4 Mb region of human chromosome 10. Top track shows Refseq genes. All other tracks display data from the human lymphoblastoid cell line GM12878. From top to bottom these tracks are: smoothed CTCF signal from ENCODE [130]; significant contact calls by Fit-Hi-C using 1 kb resolution Hi-C data (only the contacts >50 kb distance and − log(p-value) ≤25 are shown) [20]; arrowhead domain calls at 5 kb resolution [18]; Armatus multiscale domain calls for three different values of the domain-length scaling factor *γ* [87]; DI HMM TAD calls at 50 kb resolution [15]; and the heatmap of 10 kb resolution normalized contact counts for GM12878 Hi-C data [18]. The color scale of the heatmap is truncated to the range 20 to 400, with higher contact counts corresponding to a darker color

Instead of assuming a specific distribution, one can infer the distance-dependence relationship using nonparametric methods, such as splines, directly from the observed contact counts. Compared with parametric fits, nonparametric fits are more general in capturing the distance dependence, which changes substantially with varying resolution, genomic distance range, and sequencing depth [20]. A recent method, Fit-Hi-C, uses smoothing splines to find an initial fit, refines the initial fit to account for *bona fide* (non-random) contacts, and computes confidence estimates using the refined fit while incorporating biases computed by the matrix balancing-based normalization methods [20]. The resulting *p*-values are subsequently subjected to multiple testing correction. Figure 3 displays examples of long-range chromatin loops identified by Fit-Hi-C.

### Peak detection

A more recent study approaches the problem of extracting significant contacts as a two-dimensional peak detection problem [18]. The method, called HiCCUPS, computes, for each locus pair, the enrichment of its contact count with respect to various neighboring regions. For high-resolution contact maps, this enrichment calculation must be carried out on the order of 10^12^ times. To overcome this computational challenge, in addition to the CPU implementation, HiCCUPS was also implemented on GPUs. To overcome the statistical challenge of dealing with such a large number of hypotheses, HiCCUPS segregates these hypotheses into families and carries out multiple testing correction within each hypothesis family [18].

These methods attempt to distinguish between functional contacts and contacts that are due to random polymer looping or other confounding factors. Most of these methods aim to find pairs that interact much more than expected in the overall data. HiCCUPS, on the other end, is more stringent and finds only the contacts that appear as peaks in the contact maps within the surrounding region. These contacts usually correspond to precise anchoring points of highly stable chromatin loops. In either case, accomplishing the task of confidence estimation has important implications in identifying functional interactions among enhancers and promoters, and between pairs of CTCF binding sites that form chromatin loops [11, 14, 18, 20, 65].

## Testing three-dimensional colocalization of functionally associated loci

Another important benefit of having genome-wide proximity information is that it allows the testing of hypotheses related to the nuclear localizations of a given set of loci. The most common scenario is when one wants to test whether a set of loci (for example, centromeres, housekeeping genes, or DNA breakpoints) colocalize beyond ‘expected’ in three dimensions. Early methods to test whether the colocalization of a set is statistically significant used the hypergeometric approach that computes the probability of observing the number of pairwise interactions within the set among all observed pairwise interactions [8, 66]. However, Witten and Noble subsequently pointed out certain issues with the hypergeometric approach and proposed a resampling-based approach that produces uniformly distributed significance estimates when randomly generated sets of loci are used for benchmarking the statistical accuracy [67]. Witten and Noble revisited the claims made previously using hypergeometric tests and demonstrated that some of the supposedly colocalized sets of loci, such as target gene sets of certain transcription factors [66], are not colocalized more than expected when the resampling-based approach is used [67].

One limitation of all the tests described above is their inability to handle intra-chromosomal contacts. To address this shortcoming, Paulsen et al. propose a test that handles intra- and inter-chromosomal interactions, both separately and jointly [68]. This method relies on randomly selecting sets of regions that share the same structural properties as the query set. In addition to controlling for one-dimensional distance (or lack of it for inter-chromosomal contacts), Paulsen et al. develop a stricter null model that also controls for compartmental structure and the domain organization along the chromosomes. These statistical tests, together with others, are made available through a web-based tool, HiBrowse [69].

All of the hypergeometric and sampling-based approaches we have discussed so far perform the significance tests using contact counts, and, usually, by dichotomizing the pairs as ‘close’ or ‘far’ depending on the contact’s statistical significance. Capurso et al. suggest discarding this dichotomy by using pairwise distances from the three-dimensional reconstructions of chromosomes instead of contact counts [70]. However, this approach depends on the ability to generate accurate three-dimensional models, which is itself a topic of ongoing research as we elaborate below.

Whether it is the two-dimensional contact maps or the three-dimensional reconstructions used for testing spatial colocalization, it is an important task to reveal clustered elements, some of which serve as the hallmarks of genome organization such as telomeres and centromeres in yeasts [8, 28, 29, 31], virulence genes in *Plasmodium* [16], and heterochromatic islands in *Arabidopsis* [39]. Further developments in this line of computational work may allow *de novo* identification of significantly colocalized or dispersed sets of regions.

## Identifying domains in Hi-C contact maps

In the genomics literature many types of regulatory domains have been identified on the basis of specific epigenetic marks [12, 71–73], DNA replication timing [19, 21, 74], lamina associations [75, 76], nucleolus associations [77], or a joint analysis of some of these factors [78–83]. All of these domains are defined by specific patterns of one-dimensional signal tracks. With the availability of genome-wide Hi-C data, several novel domain types have been identified that appear as specific patterns in contact maps. These include open/closed chromatin compartments identified by eigenvalue decomposition [7, 47], subcompartments of these open/closed compartments identified by clustering [18], and topologically associated domains (TADs) identified as densely interacting squares on the diagonal of the contact map [15, 84]. TADs are of particular interest recently, and a variety of methods have been developed to identify and characterize these domains. Below we briefly discuss these methods to identify TADs from Hi-C data. For further discussion of other domain types, see [63, 85, 86].

### Directionality Index Hidden Markov Model (DI HMM)

A TAD creates an imbalance between the upstream and downstream contacts of a region. This imbalance is an indicator of whether a region is in the inside, at the boundary, or far away from a TAD. Dixon et al. quantify this imbalance in a statistic named directionality index (DI) and use an HMM to determine the underlying bias state for each locus (upstream, downstream, none) [15]. They then use these HMM state calls to infer TADs as continuous stretches of downstream bias states followed by upstream bias states. A region in between two TADs is either called a boundary or unorganized chromatin depending on the region’s length. Other studies also use directionality bias-based statistics to determine domain presence and domain coordinates in mitotic human cells [43] and in fission yeast [32].

### Domain borders as peaks of the distance-scaling factor

TADs also create unexpectedly low numbers of contacts crossing the boundary regions. Sexton et al. use this property to infer a distance-scaling factor for each restriction fragment, which is high if the fragment insulates its upstream regions from the downstream, effectively acting as a much longer fragment than its actual size [30]. The peaks in these distance-scaling factors then correspond to boundaries of what they call physical domains for the *Drosophila melanogaster* genome.

### Multiscale and hierarchical domains

It is clear from visual inspection of contact heatmaps that there are sub-structures within TADs that may also correspond to hierarchical units of gene regulation or other functions. Filippova et al. propose a dynamic programming method called ‘Armatus’ to identify optimal and near-optimal domains for a given resolution [87]. From the resulting sets of resolution-specific domains, they then identify a consensus set that consists of the domains that are consistent across different resolutions. Both the resolution-specific domains and the consensus domains are then used as TAD calls for downstream analysis. Another dynamic programming method, HiCseg, computes the optimal segmentation into TADs via a maximum likelihood formulation [88]. However, HiCseg does not readily allow identification of multiscale or hierarchical domains.

### Arrowhead algorithm

To make use of very high resolution contact maps, Rao et al. propose a heuristic method to find the corners of domains in the human and mouse genomes that are 4-5 times smaller than previously identified TADs [18]. This method first transforms a contact map to an arrowhead matrix in which each entry *A*_*i*,*i*+*d*_ corresponds to the directionality bias of locus *i* at only the exact distance *d*. This matrix results in arrowhead shaped patterns at the corners of domains. Rao et al. then heuristically search for these arrowhead patterns using criteria derived from known TADs.

Figure 3 plots the TAD calls from three of the above methods for an approximately 1 Mb locus on chromosome 10 using Hi-C data for the human GM12878 cell line. Some of these methods find substantially different numbers of TADs with different length distributions compared to the others. This difference is partly due to the differences in the resolutions of the contact maps used or the length of the flanking regions considered in the algorithms (see [18] and [87] for comparisons of Arrowhead algorithm and Armatus with DI HMM). However, these differences also indicate that using a single set of non-overlapping domains may be a simplification, both because of the potential heterogeneity of domain organization in the underlying cell population and because of the hierarchical and dynamic organization of chromatin that allows efficient folding and unfolding. For further information on why TAD organization and its changes are important in gene regulation and genome function, see [89–91].

## Three-dimensional modeling of chromatin structure

In the absence of chromatin conformation capture data, three-dimensional modeling of genome architecture can be carried out using polymer physics simulations that rely on a limited number of physical assumptions and parameters. Rosa et al. refer to such polymer models as ‘direct’ models of genome architecture, because they do not rely on indirect measurements of chromatin structure such as Hi-C [92]. These polymer approaches represent chromosomes as self-avoiding polymer chains that move within the constrained nuclear space. Some of these approaches use Hi-C data to validate their inferred structures for well studied genomes such as budding yeast [93–97]. Detailed discussions of the various polymer models in the context of genome architecture, which is beyond the scope of this review, can be found in several review articles [90, 92, 98].

With the availability of genome-wide contact maps, the reconstruction of the three-dimensional chromatin structure that underlies the observed contacts became a fundamental problem. These observed contact maps made it possible to generate detailed three-dimensional models using the contact counts as soft ‘restraints’ (in contrast to hard constraints) on the relative locations of loci with respect to each other. Fittingly, these models are referred to as restraint-based models [90, 99]. Other terms used for these models include probabilistic, statistical, or ‘inverse’ models, in contrast to polymer-based direct models [92]. These restraint-based models can be further divided into two groups. The first group of methods aim to find a consensus three-dimensional conformation that best describes the observed Hi-C data. However, the standard Hi-C protocol pools millions of cells for library creation (bulk); therefore, the readout represents a mixture of potentially different conformations. To account for this cellular heterogeneity, the second group of methods, instead infer an ensemble of structures from the bulk Hi-C data. Both of these approaches, consensus and ensemble, have given rise to reconstruction methods that have been reviewed previously [50, 90, 92, 98–102] and are also briefly outlined below.

### Consensus methods

One of the most commonly used methods to infer consensus three-dimensional models from conformation capture data is multi-dimensional scaling (MDS) [8, 16, 31, 101, 103–106]. MDS is a classical statistical method that, given all pairwise distances between a set of objects, aims to find an N-dimensional embedding such that the pairwise distances are preserved as well as possible [107]. In this context, objects are beads that represent chunks of DNA, and pairwise distances are computed by applying a transfer function on contact counts. Several studies use metric MDS augmented with additional constraints on the polymer characteristics, hence intersecting with polymer models, or on the genome organization (such as clustering of centromeres) to find a consensus structure [8, 16, 31]. With or without these additional constraints, the MDS formulation gives rise to a non-convex optimization problem requiring heuristic optimization methods such as gradient descent, conjugate gradient, and simulated annealing. A recent method applies a semidefinite programming (SDP) approach to three-dimensional genome reconstruction [103]. This method uses a relaxation of the solution space of each bead from $\mathbb {R}^{3}$ to $\mathbb {R}^{n}$, where *n* is the number of beads, to transform certain MDS formulations into convex semidefinite programs. The SDP approach guarantees perfect three-dimensional reconstruction if the input pairwise distances are noise-free. However, a major drawback of SDP, as opposed to classical MDS-based solutions, is computational expense on datasets with realistic resolutions. Furthermore, all MDS-based methods depend on a transfer function that converts contact counts to pairwise spatial distances, and the methods are very sensitive to the selection of this transfer function [101, 103]. Several methods use non-metric MDS that avoids any assumptions about the transfer function and calculates the count-to-distance relationship through isotonic regression [101, 104]

### Ensemble methods

For inference of an ensemble of three-dimensional models, several probabilistic methods have been proposed that produce a set of structures representative of the observed contact data. These methods can be further divided into two depending on whether they aim to find multiple solutions, each of which fits the bulk Hi-C data, or to find a ‘true’ ensemble that, in aggregate, optimally describes the bulk data. The first case is similar to the consensus approach, but instead of inferring one locally optimal model, the optimization is run with multiple initializations resulting in multiple different models [105]. The variability among these models depends heavily on the problem structure and on the random initializations, making it difficult to link the resulting models to the cellular variability of chromatin structure in the bulk sample. Rousseau et al. develop a similar method that uses Markov Chain Monte Carlo (MCMC) sampling to approximate the posterior probability of each model given the data from a large number of models that are independent of random initialization [108]. Giorgetti et al. use a very similar MCMC-based approach for ensemble modeling of mouse chromosomes [109]. The second case is more challenging because it requires coordinated inference of a large number of models. Hu et al. use MCMC with a mixture model component to determine whether a mixture of structures better explain the conformation of a locus than a single consensus structure [110]. Kalhor et al., on the other hand, develop a method that truly mimics the bulk nature of the Hi-C experiment [9]. They simultaneously infer, in a single optimization, thousands of structures, each of which are fully consistent with the constraints derived from the bulk data and which, in aggregate, best explain the bulk contact counts. Many other ensemble methods have been developed in the past 3 years [102, 111, 112] to characterize the cell-to-cell variability of chromatin structure in the bulk Hi-C data. Furthermore, Nagano et al. demonstrate the feasibility of generating single-cell Hi-C data, leading to a more direct characterization and modeling of the cellular variation of chromosome structure [24].

## Visualization of Hi-C data

Visualization of genomics data is crucial for both hypothesis generation and detection of potential artifacts. Several genome and epigenome browsers are used heavily for visualizing thousands of data tracks for human, mouse and other organisms [113–116]. However, these browsers are mainly designed for visualization of one-dimensional signals and are not easily extensible to visualizing two-dimensional Hi-C or any conformation capture data. Furthermore, as we discussed above, Hi-C data can be used for three-dimensional modeling, which requires tools not only for two-dimensional but also for three-dimensional visualization.

To address this need, several existing tools, such as the WashU Epigenome Browser, now allow browsing of long-range contact data [117]. Figure 3 shows a snapshot from this browser in which one-dimensional data tracks are overlaid with contact information from Hi-C data as either long-range arcs or rotated heatmaps. Certain one-dimensional aspects of Hi-C data, such as the total contact count per locus, principal components, directionality of contact preference, and topological domain boundaries, can also be overlaid with other data. Another visualization tool, the Hi-C Data Browser [118], uses the UCSC Genome Browser [113] to allow simultaneous viewing of rotated Hi-C heatmaps and UCSC tracks. A more recent desktop application, Juicebox, allows users to view heatmaps of multiple human and mouse Hi-C datasets together with other features such as domain calls, peak calls from HiCCUPS, and CTCF binding sites [18]. Several tools are currently under development for visualization of three-dimensional models of chromatin, including Genome3D [119] and TADkit [120].

## Outlook

We have discussed here the major steps in analyzing Hi-C datasets and outlined currently available computational tools and methods to perform each step. Although the diversity of available methods provides alternative ways to explore Hi-C data, it is becoming clear that converging to a common set of tools will be useful to compare and consolidate results from the increasing number of publications. We also believe that reaching a similar consensus on the quality control metrics and the terminology used for Hi-C data will be beneficial for the field. For instance, the term ‘normalization’ may refer to the correction of sequencing-related factors in Hi-C contact counts [18, 47] or to the correction of genomic distance effect [62, 121]. Similarly, multiple different terms, such as TADs [15, 84], physical domains [30], and loop domains [18], may refer to a single type of pattern observed in contact maps.

On the other hand, this diverse set of computational methods falls short of fully exploiting the power of Hi-C data. For instance, very few tools performcomparative analysis, visually or statistically, of two Hi-C contact maps [59, 62, 69, 122], and none of these tools allow joint analysis of more than two datasets that come from multiple time points, conditions, or cell types. Also, many of the existing methods, specifically the three-dimensional reconstruction algorithms, do not scale to high-resolution Hi-C data from large genomes such as human and mouse. Deconvolution of Hi-C data from a large number of cells into subpopulations with similar chromatin organizations and estimation of the density of each subpopulation is still largely unexplored [123, 124]. Similarly, integration of two-dimensional Hi-C data or three-dimensional chromatin models with the vast quantity of available one-dimensional datasets, such as replication timing, histone modifications, protein binding and gene expression, is also understudied. One study that integrates Hi-C data with many types of genomics and epigenomics data tracks uses a technique called graph-based regularization (GBR) to perform semi-automated genome annotation [86]. This study encouragingly shows that the integration of Hi-C data improves the annotation quality and allows identification of novel domain types. However, GBR assumes that regions that are close in three dimensions should be assigned the same annotation label, which may only makes sense for large-scale domain annotations (greater than approximately 100 kb). Another method integrates low resolution Hi-C data (1 Mb) with transcription-factor binding, histone modification and DNase hypersensitivity information and identifies 12 different clusters of interacting loci that fall into two distinct chromatin linkages (co-active and co-repressive) [125]. Most recently, Chen et al. present a unified four-dimensional analysis framework (three space plus one time dimension) that uses adaptive resolution contact maps to perform gene-level analysis [44]. They use this framework to interrogate the dynamic relationship between genome architecture and gene expression of primary human fibroblasts over a 56-hour time course. Concurrent advances in such computational integration efforts and in experimental data generation have the potential to transform our understanding of the structure-function relationship and help translational biomedical research. Several intriguing studies suggest that alterations in chromatin conformation and in gene regulation are tightly linked in cancer [22, 23, 126, 127], cellular differentiation [128], and development [129].

Other challenges in the field that require partly computational and partly experimental advances are: (i) characterizing the cell-to-cell variability of chromatin structure using large numbers of single cells, (ii) inferring haplotype-specific contact maps and three-dimensional chromosome structures, and (iii) distinguishing direct DNA-DNA contacts between two loci from indirect, bystander, or protein-mediated interactions. Recent advances in technology development suggest that we are not far away from overcoming the experimental bottlenecks surrounding the above-mentioned challenges [17, 18, 24]. Therefore, it is essential to forge ahead with the development of computational methods that are both theoretically sound and practically scalable, in preparation data.
